# Predicting disease associations based on the higher order structure of ceRNA networks

**DOI:** 10.1093/bib/bbaf518

**Published:** 2025-10-04

**Authors:** Zhaoliang Chai, Ying Su, Xuecong Tian, Chen Chen, Xiaoyi Lv, Cheng Chen

**Affiliations:** College of Computer Science and Technology, Xinjiang University, Urumqi 830046, Xinjiang, China; College of Computer Science and Technology, Xinjiang University, Urumqi 830046, Xinjiang, China; College of Computer Science and Technology, Xinjiang University, Urumqi 830046, Xinjiang, China; College of Software, Xinjiang University, Urumqi 830046, Xinjiang, China; Xinjiang Cloud Computing Application Laboratory, Karamay 834000, Xinjiang, China; Xinjiang Hongyou Software Inc., Karamay 834000, Xinjiang, China; College of Software, Xinjiang University, Urumqi 830046, Xinjiang, China; The Key Laboratory of Signal Detection and Processing, Xinjiang University, Urumqi 830046, Xinjiang, China; College of Software, Xinjiang University, Urumqi 830046, Xinjiang, China

**Keywords:** ceRNA network, disease prediction, graph attentionnetwork, graph convolutional neural network

## Abstract

Competitive endogenous RNA (ceRNA) network regulation is an important posttranscriptional regulatory mechanism that plays an important role in physiological and pathological processes, and has been widely used in biomarker screening and regulatory factor studies of disease-related genes. However, existing studies have mainly focused on the association of a single type of RNA with disease, while studies targeting the application of ceRNA networks in disease prediction are still limited, so it is crucial to explore the potential of ceRNA networks in disease prediction. In this study, we propose CERDA-HOSR, a computational method for mining ceRNA network–disease associations based on higher order graph attention networks. The method uses higher order graph convolutional networks to aggregate neighborhood information to generate representations of different RNAs and diseases. Given the higher order complexity of biological networks and sample imbalance problem, traditional random negative sampling is difficult to effectively capture global information; for this reason, a higher order negative sampling strategy is designed to optimize the quality of negative samples by combining the network structure and higher order neighborhood relations to improve the generalization ability and prediction accuracy of the model. Finally, LightGBM calculates the ceRNA network–disease association probability based on the learned embedding. A large number of simulation experiments validate the superiority of CERDA-HOSR, and its practical application is further demonstrated by case studies of cardiovascular disease, acute myeloid leukemia, and papillary thyroid cancer. In addition, ablation experiments and exploratory analyses further enhance its robustness and provide an effective tool for disease prediction and biomarker screening.

## Introduction

In recent years, competing endogenous RNA (ceRNA) networks have emerged as a research hotspot in the biomedical field, attracting increasing attention for their roles in gene expression regulation. By enabling RNA molecules to competitively bind to microRNAs (miRNAs), ceRNA networks influence the expression of target genes and offer a novel perspective for transcriptomic research [1]. The ceRNA network involves various types of RNA molecules, such as messenger RNA (mRNA), long noncoding RNA (lncRNA), circular RNA (circRNA), and pseudogenes [2, 3], and provides a more comprehensive representation of complex biological regulatory processes compared with traditional miRNA networks [4].

In ceRNA networks, common regulatory pathways include circRNA–miRNA–mRNA and lncRNA–miRNA–mRNA axes. MicroRNAs occupy a central position in these networks. When miRNAs are competitively bound by lncRNAs or circRNAs, their repressive effects on target mRNAs may be attenuated, leading to increased mRNA expression levels. However, the ceRNA regulatory mechanism is not universally applicable; effective competitive interactions can only occur when the involved RNA molecules share common miRNA response elements. This competitive binding mechanism enables endogenous RNAs to regulate each other’s expression and is known as the ceRNA hypothesis [5, 6]. This hypothesis was first validated in *Arabidopsis thaliana* [7] and has been shown to play a significant role in complex diseases such as cancer [8, 9].

Currently, ceRNA networks have attracted considerable attention in disease mechanism research. However, their systematic exploration in disease prediction remains limited. Most existing approaches focus on a single type of RNA molecule (such as lncRNA or miRNA), overlooking the integrative potential of ceRNA networks across multiple RNA species. Moreover, challenges such as data sparsity, multi-omics data integration, and the extraction of high-dimensional nonlinear features remain to be addressed. Therefore, further exploration of the complex associations between ceRNA networks and diseases, along with the development of efficient computational prediction models, will not only advance ceRNA research but also provide new support for precision medicine and targeted therapy [10, 11].

In recent years, an increasing number of studies on disease prediction have begun to integrate multiple RNA types—such as miRNA, lncRNA, and circRNA—to construct more comprehensive regulatory networks and enhance the accuracy of predictive models [12, 13]. For example, the MAGCN method [14] leverages graph convolutional networks (GCNs) [15] to integrate lncRNA–miRNA interaction data and employs a multichannel attention mechanism to accurately identify miRNA–disease associations, thereby avoiding biases caused by traditional similarity-based feature selection. In another study, transcriptomic data from breast cancer and bladder cancer were jointly analyzed to construct mRNA/lncRNA classifiers, which were further extended into ceRNA networks to investigate shared molecular characteristics between the two cancers [16]. Meanwhile, in cardiovascular disease research, researchers have constructed regulatory sub-networks based on interactions among circRNA, miRNA, and mRNA to identify potential disease biomarkers and pathogenic pathways [17, 18].

With the rapid development of graph neural networks (GNNs) and their variants, these models have been widely applied in RNA–disease association prediction due to their powerful capabilities in structural modeling and integrating high-dimensional data from heterogeneous networks. By constructing biomolecular graph models, GNNs can effectively capture complex relationships among heterogeneous molecules and high-dimensional topological structures, thereby enhancing the identification of disease biomarkers. However, most existing methods primarily focus on direct binary interactions and have yet to fully exploit high-order structural features embedded in ceRNA networks. Such features are crucial for characterizing the multilayered regulatory relationships between RNAs and diseases. Effectively incorporating high-order structural information into predictive models remains a key challenge that requires urgent attention.

Due to the time-consuming and costly nature of laboratory experiments, computational approaches have become the mainstream for RNA–disease association prediction [19], primarily including traditional machine learning methods [20] and deep learning-based approaches [21]. Compared with traditional methods, deep learning captures complex relationships and is widely used in tasks such as action recognition [22, 23], link prediction [24], knowledge graph [25], and graph representation learning [26], and has shown advantages in the prediction of associations between RNA-diseases.

Most of the existing models are variants of GCN, GAT, and GAE. For example, LAGCN [27] combines GCN with attention mechanism for drug–disease prediction; MMGCN [28] predicts miRNA-disease associations using a multichannel attention mechanism, GCNAT fuses GCN and GAT to enhance metabolite-disease prediction, and VGAE-MDA [29] integrates miRNA and disease information via variogram autoencoder. In addition, methods such as AEMDA [30] and DFELMDA [31] combine deep autocoders with random forests to improve prediction accuracy, while GAEMDA [32] uses GNN encoders to obtain node embeddings and completes the prediction with a bilinear decoder. Meanwhile, researchers have also attempted to construct heterogeneous networks to capture the complex associations among different types of molecules. For instance, HINLMI [33] employs a heterogeneous information network to extract molecular features and predict lncRNA–miRNA associations, while GCLMTP [34] utilizes a graph contrastive learning model to explore interaction patterns among lncRNA, miRNA, and diseases. These approaches offer novel perspectives for RNA–disease association studies [35–39].

Despite the progress achieved in specific tasks, existing studies still face several limitations: (i) insufficient RNA type coverage: many approaches overlook noncoding regulatory elements such as circRNAs and lncRNAs, thus failing to fully exploit the integrative potential of ceRNA networks; (ii) structural modeling limitations: most models focus on single-layer interactions and lack the capacity to capture multi-order regulatory pathways; (iii) label leakage and false negatives: issues such as label leakage and underexplored false negative samples remain unresolved, which compromises the generalization ability of predictive models.

To address the aforementioned challenges, we propose CERDA-HOSR (ceRNA–Disease Association with Higher-Order Structure Representation), a novel model that constructs a unified heterogeneous hypergraph representation. By incorporating a dynamic reconstruction mechanism while preserving structural properties, the model significantly enhances the ability to capture both semantic and topological features among different RNA types, thereby enabling more efficient and accurate disease prediction from multiple RNA perspectives. The main contributions of this study are summarized as follows: (i) high-order information modeling: we capture the multilevel regulatory features among lncRNAs, miRNAs, and mRNAs, and extract higher order topological information associated with ceRNA–disease interactions. (ii) Integration of multilayer GCN and high-order GAT: the GCN component aggregates features from both direct and higher order neighbors, while the high-order GAT refines attention allocation across nodes to more precisely model complex regulatory relationships. (iii) High-order negative sampling strategy: a structure similarity–based sampling approach is designed to generate high-quality negative samples among homogeneous nodes, which enhances the model’s robustness and generalization performance.

## Materials and methods

**Figure 1:**
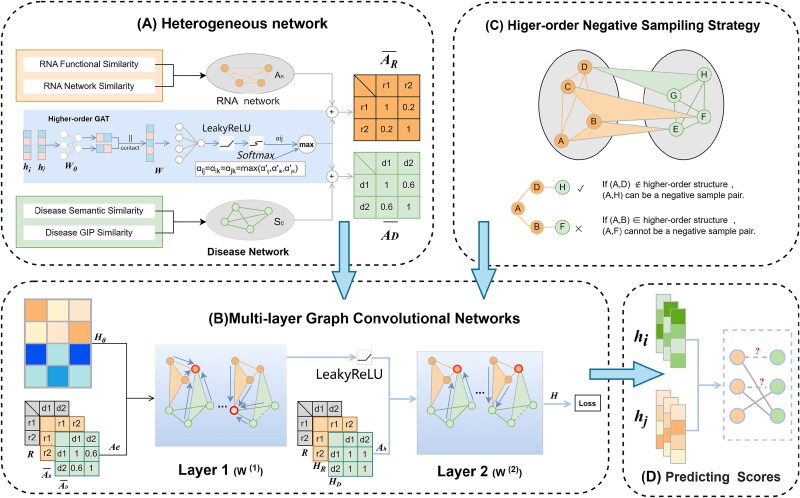
Higher order structure-based ceRNA network–disease prediction framework.

This section provides a detailed description of the similarity computation methods for different RNAs and diseases, the design of the high-order GAT, multilayer GCN, and the high-order negative sampling strategy. The overall architecture of the CERDA-HOSR framework is illustrated in Fig. 1.

Figure 1 illustrates the overall framework of the proposed prediction model. (A) **Fusion similarity computation**: the similarity matrices of RNAs and diseases are fused by adding them to the corresponding attention coefficients, resulting in two integrated similarity matrices, $A_{R}$ and $A_{D}$. (B) **Node embedding update**: node embeddings are updated using a two-layer high-order GCN with different adjacency matrices, $A_{e}$ and $A_{h}$, generating embedding vectors $h_{\text{lnc}}$, $h_{\text{mi}}$, $h_{\text{mr}}$, and $h_{\text{disease}}$ for the four types of nodes. (C) **High-order negative sampling strategy**: a negative sampling strategy is designed based on the assumption that homogenous nodes are more similar in feature space than randomly sampled pairs, thereby enhancing training effectiveness. (D) **Prediction probability computation**: the final node embeddings are fed into a LightGBM (LGBM) model to predict the probability of ceRNA–disease associations.

### RNA similarity

In this study, RNA functional similarity and network-based similarity [40] are primarily employed for similarity computation. Based on RNA functional similarity, MISIM3 calculates the functional similarity between miRNAs under the assumption that functionally similar miRNAs are often associated with similar diseases [41]. The functional similarity between RNA *i* and *j* is denoted as *FS*(*i*, *j*). However, since existing functional similarity data only cover a subset of miRNAs, we further incorporate a network structure-based similarity computation method to complement the similarity information for lncRNAs, miRNAs, and mRNAs.

In ceRNA network analysis, the Node2Vec algorithm is an effective approach for computing the similarity between different nodes by leveraging node embeddings to capture both topological structures and semantic features. Node2Vec is an efficient network embedding algorithm that learns low-dimensional representations of nodes by simulating biased random walks to generate node contexts. Specifically, Node2Vec captures both local neighborhoods (micro-level structures) and global relationships (macro-level structures) in the network, generating embedding vectors for each node that encode their topological and semantic characteristics. The similarity between lncRNA, miRNA, and mRNA nodes is then evaluated by computing the similarity between their corresponding embedding vectors.

Let the ceRNA network be represented as $\mathit{G} = (\mathit{V}, \mathit{E} )$, where *V* denotes the set of nodes—including $V_{\text{lncRNA}}$, $V_{\text{miRNA}}$, $V_{\text{mRNA}}$, and $E$ represents the set of edges, indicating the interactions between nodes. The process of generating node embeddings is described as follows:

(1) **Random walk strategy**: the core idea of Node2Vec lies in generating training data through biased random walks, where the walk behavior can be tuned between breadth-first and depth-first search via two parameters, *p* and *q*. Starting from a node *v*, the probability of transitioning to the next node is defined as
  (1) \begin{align*}& P(c \mid v) = \begin{cases} \frac{\pi_{vc}}{z}, & \text{if}\ (v, c) \in E \\ 0, & \text{ otherwise} \end{cases}\end{align*}
  Here, $ \pi _{vc} $ represents the transition weight from node $ \mathit{v} $ to node $ \mathit{c} $, and $ \mathit{Z} $ is the normalization constant. The transition weight for edge ($ \mathit{v} $, $ \mathit{c} $) is influenced by the parameters $ \mathit{p} $ and $ \mathit{q} $, and is defined as follows:
  (2) \begin{align*}& \pi_{vc} = \begin{cases} \frac{1}{p}, & \text{if}\ d(t, c) = 0 \text{ (return to previous node)} \\ 1, & \text{if}\ d(t, c) = 1 \text{ (go to neighborhood node)} \\ \frac{1}{q}, & \text{if}\ d(t, c) = 2 \text{ (go to distant neighbor node)} \end{cases}\end{align*}
  Here, *d* (*t*,*c*) denotes the shortest-path distance between the previous node *t* and the current candidate node *c*. The random walk path is represented as follows: $\mathit{R}(\mathit{v}) = \{ \mathit{v}_{1}, \mathit{v}_{2}, \dots , \mathit{v}_{l} \}, \mathit{v}_{i} \in V$, where $\mathit{v}_{1}$ is the starting node, and $l$ is the length of the walk.
(2) **Node embedding and skip-gram optimization**: Node2Vec optimizes the random walk paths using the Skip-gram model, aiming to maximize the conditional probability of a node given its context, thereby learning low-dimensional vector representations of nodes. Specifically, for a node $v$ and its context node set $N_{r}(v)$, the objective is to maximize the conditional probability of observing a context node $u$ given the target node $v$, defined as:
  (3) \begin{align*}& Z_{v} = \prod_{u \in N_{r}(v)} P(u \mid Z_{v})\end{align*}
  Here, the conditional probability $P(u|Z_{v})$ is computed using the $Softmax$ function:
  (4) \begin{align*}& P(u \mid Z_{v}) = \frac{\exp ( z_{u} \cdot z_{v} )} {\sum\limits_{w \in V} \exp ( z_{w} \cdot z_{v} )}\end{align*}
  Node2Vec applies the Skip-gram model to each node sequence $R(v)$, aiming to optimize the conditional probability between the target node $v$ and its context nodes $u$. The objective function is defined as:
  (5) \begin{align*}& L = \sum\limits_{v \in V} \sum\limits_{u \in N_{r}(v)} \log P(u \mid Z_{v})\end{align*}
  To reduce computational complexity, Node2Vec adopts a negative sampling strategy. The objective function is approximated by optimizing the following expression:
  (6) \begin{align*}& L = \log \sigma (z_{u} \cdot z_{v}) + \sum_{k=1}^{K} \mathbb{E}_{v_{n} \sim P_{n}} \left[ \log \sigma (- z_{v_{n}} \cdot z_{v}) \right],\end{align*}
  where $\sigma (x) = \frac{1}{1+\exp (-x)}$ is the *Sigmoid* function, $\mathit{v}_{\mathit{n}}$ is a negatively sampled noise node, the distribution $\mathit{P}_{\mathit{n}}$ is usually taken as $\mathit{P}_{\mathit{n}}(\mathit{v}) \propto d(v)^{3/4}$, and $\mathit{d}(\mathit{v})$ is the degree of the node $\mathit{v}$.
(3) **Node similarity calculation**: after obtaining the embedding representations of nodes, a similarity matrix *S* is constructed, where each element $S_{ij}$ denotes the similarity between nodes $v_{i}$ and $v_{j}$. This similarity is computed using cosine similarity as follows:
  (7) \begin{align*}& Sim(u, v) = \frac{z_{u} \cdot z_{v}}{\| z_{u} \| \| z_{v} \|},\end{align*}
  where $z_{u}$ and $z_{v}$ are the embedding vectors of nodes $u$ and $v$, respectively; $\| z_{u} \|$ and $\| z_{v} \|$ are the Euclidean norms of the corresponding vectors.
By integrating random walks with the Skip-gram model, Node2Vec effectively captures both local and global relationships among nodes and generates low-dimensional embedding vectors. Its flexibility and efficiency offer significant advantages in analyzing node relationships within complex biological networks, making it particularly well-suited for handling multiple node types and their interactions in ceRNA networks.

### Disease similarity

**Figure 2:**
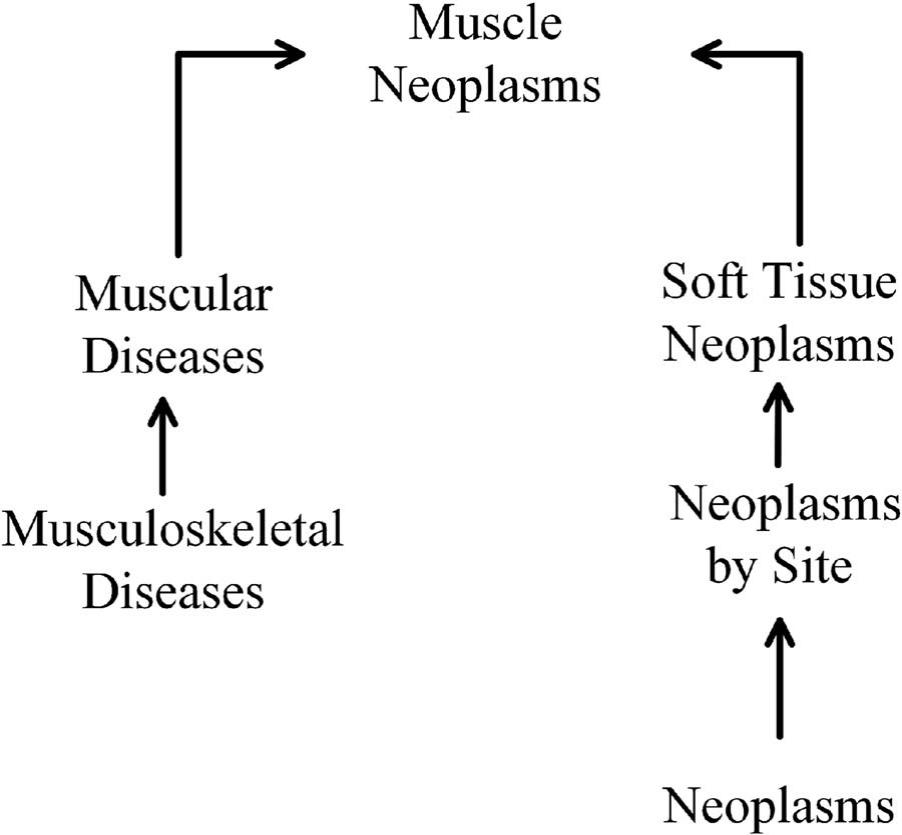
The $DAG$ of Muscle Neoplasms.

The calculation of semantic similarity of diseases is based on the methodology in the literature [42]. This study incorporated two primary datasets, Disease Ontology (DO [43]) and Medical Subject Headings (MeSH [31]), to analyze semantic relationships in diseases. These datasets structure disease associations within a directed acyclic graph (*DAG*) framework. For each disease *d*, its *DAG* form is denoted as $DAG_{d}$ = ($N_{d}$, $E_{d}$), where $N_{d}$ represents disease *d* and the set of all ancestor nodes, and $E_{d}$ represents the set of edges between nodes. Take the muscle tumor in Fig. 2 as an example.

If a $DAG_{d}$ exists for disease $X$, its contribution to the semantic similarity of a disease can be quantified following the approach outlined in [42]:


(8) \begin{align*}& D_{d}(X) = \begin{cases} 1, & \text{if}\, X = d \\ \max \{\mu \cdot D_{d}(X^{\prime}) \mid X^{\prime} \in \text{children of}\ X \}, & \text{if}\, X \neq d \end{cases}\end{align*}

Here, $\mu $ is the semantic value contribution factor of disease $X$ to disease $X^{\prime}$. In the article [44], $\mu $=0.5. Therefore, the semantic value $DV(d)$ of disease $d$ can be expressed as [42]


(9) \begin{align*}& DV(d) = \sum_{X \in N_{d}} D_{d}(X).\end{align*}

It can be seen that the more shared components diseases $i$ and $j$ have in the $DAG$, the higher their semantic similarity. The semantic similarity [42] between disease $i$ and $j$ can be defined as


(10) \begin{align*}& DS(i, j) = \frac{\sum\limits_{X \in N_{i} \cap N_{j}} (D_{i}(X) + D_{j}(X))}{DV(i) + DV(j)}\end{align*}

However, for some diseases for which semantic information is not available, this study uses the GIP kernel similarity method [44]. For ease of representation, we chose the 0-1 vector as the interaction distribution for disease $i$. The GIP kernel similarity $GS_{1}(i,j)$ for diseases $i$ and $j$ can be expressed as [44]


(11) \begin{align*}& GS_{1}(i,j) = \exp \left( - \Delta_{d} \| \beta(d_{i}) - \beta(d_{j}) \|^{2} \right),\end{align*}

where $\beta (d_{i})$ and $\beta (d_{j})$ denote the interaction distributions of diseases $i$ and $j$, respectively, and each row of the RNA–disease association matrix $R$ denotes the association of a particular RNA with a disease. An association is labeled as 1 if it exists, and 0 otherwise. Vectors $\beta (d_{i})$ and $\beta (d_{j})$ correspond to row $i$ and column $j$ of $R$, respectively. Similarly to literature [44], $\Delta _{d}$ is defined as


(12) \begin{align*}& \Delta_{d} = \frac{1}{\frac{1}{n} \sum\limits_{i=1}^{n} \|\beta(d_{i})\|^{2}},\end{align*}

where $n$ is the total number of diseases. The GIP kernel similarity of the diseases was obtained by the above method. In summary, the semantic similarity $DS(i,j)$ (i.e. Equation (10)) is used for diseases with semantic information, and the GIP kernel similarity $GS_{1}(i,j)$ (i.e. Equation (11)) is used for diseases without semantic information.

### RNA–disease heterogeneous networks

In the RNA–disease network, $V$ denotes the set of nodes, including both RNAs and diseases, and $E$ represents the set of associations between these nodes. In the RNA–disease association matrix $R$, if there is an association between RNA $i$ and disease $j$, then $R(i,j)$ = 1; otherwise, $R(i,j)$ = 0. Subsequently, a heterogeneous network consisting of RNA and disease nodes is constructed, and its adjacency matrix is represented as


(13) \begin{align*}& \begin{aligned} R&= \begin{bmatrix} A_{\ln c-\ln c} & R_{\ln c-mi} & R_{\ln c-mr} & R_{\ln c-dis} \\ R_{\ln c-mi}^{T} & A_{mi-mi} & R_{mi-mr} & R_{mi-dis} \\ R_{\ln c-mr}^{T} & R_{mi-mr}^{T} & A_{mr-mr} & R_{mr-dis} \\ R_{\ln c-dis}^{T} & R_{mi-dis}^{T} & R_{mr-dis}^{T} & A_{dis-dis} \end{bmatrix}\in R^{N_{v}\times N_{v}},\end{aligned}\end{align*}

where $R_{lnc-mi}$ denotes the association matrix between lncRNAs and miRNAs, and $A_{dis-dis}$ represents the similarity matrix between lncRNAs. In the same way, other relationships association matrices can be obtained.

### Higher order graph attention networks

Conventional graph attention networks (GATs) [45] do not fully account for the influence of higher order structures in real-world applications. However, higher order structures are crucial for computing attention coefficients between nodes, as they can significantly enhance the model’s ability to capture complex relational patterns and improve learning performance. Ignoring such structural information may limit the effectiveness of GATs in capturing latent associations and global characteristics of the network.

In this study, to more accurately model the relationships between nodes $i$ and $j$ based on higher- order structures, the attention coefficient is computed as follows [46]:


(14) \begin{align*}& \alpha_{ij}^{\prime}=\frac{\exp\left(\mathit{Leaky\ ReLU}(W[W_{0}\vec{h_{i}}||W_{0}\vec{h_{j}}])\right)}{\sum_{b\in N_{i}}\exp\left(\mathit{Leaky\ ReLU}(W[W_{0}\vec{h_{0}}_{i}||W_{0}\vec{h_{j}}])\right)},\end{align*}

where $W$ and $W_{0}$ denote trainable weight matrices, which are dimensionally transformed by multiplying them with vectors; $\vec{h_{i}}$ represents the initial randomly generated feature of node $i$. In Equation (14), a $Softmax$ operation is applied to normalize the attention coefficients. The resulting $\alpha ^{\prime}_{ij}$ denotes the attention coefficient between node $i$ and node $j$. For ordinary node pairs that do not form higher order structures, their attention coefficients are still computed using Equation (14).

In particular, for nodes forming higher-order structures, the attention coefficients between them — denoted as $\alpha _{ij},\alpha _{ik}, \text{and} \ \alpha _{jk}$ correspond to the highest value among them, mathematically expressed as


(15) \begin{align*}& \alpha_{ij} = \alpha_{ik} = \alpha_{jk} = \max\{\alpha^{\prime}_{ij}, \alpha^{\prime}_{ik}, \alpha^{\prime}_{jk}\}.\end{align*}

After obtaining the attention coefficients between nodes, these coefficients are used to further enhance the original adjacency matrix, as defined in Equation (13).

### Multilayer GCN

In this study, a two-layer GCN is designed. The first layer is responsible for aggregating information from immediate neighbors based on the fused similarity, while the second layer focuses on aggregating higher order neighborhood features. This process is accomplished by constructing two different adjacency matrices, $A_{e}$ and $A_{h}$, corresponding to the immediate and high-order connections, respectively.

In the first layer of the multilayer GCN, to enhance the performance and efficiency of the algorithm, node similarity is introduced as a weighting factor in the computation of attention coefficients. Specifically, by integrating similarities among different types of nodes, an enhanced similarity metric is constructed. After incorporating the attention coefficient $\alpha _{ij}$, the resulting matrices $\widetilde{A}_{R}$ and $\widetilde{A}_{D}$ are defined as the fused similarity matrices, which are used to comprehensively capture the relationships between nodes.If nodes $i$ and $j$ belong to the same type of RNA nodes, their similarity is defined by $\widetilde{A}_{R}$ as follows:


(16) \begin{align*}&\widetilde{A}_{R}(i,j)=A_{R}(i,j)+\alpha_{ij}\end{align*}

For disease nodes $i$ and $j$, similarity is characterized as


(17) \begin{align*}&\widetilde{A}_{D}(i,j)=A_{D}(i,j)+\alpha_{ij}\end{align*}

This design effectively improves the accuracy of the network in capturing high-order topological relationships while maintaining high computational efficiency. Finally, the enhanced adjacency matrix $A_{e}$ can be expressed as


(18) \begin{align*}&A_{e} = \begin{bmatrix} \widetilde{A}_{lnc} & R_{lnc-mi} & R_{lnc-mr} & R_{lnc-dis} \\ R_{lnc-mi}^{T} & \widetilde{A}_{mi} & R_{mi-mr} & R_{mi-dis} \\ R_{lnc-mr}^{T} & R_{mi-mr}^{T} & \widetilde{A}_{mr} & R_{mr-dis} \\ R_{lnc-dis}^{T} & R_{mi-dis}^{T} & R_{mr-dis}^{T} & \widetilde{A}_{dis} \end{bmatrix}\end{align*}

The higher order adjacency matrix $A_{h}$ is defined similarly to $A_{e}$, aiming to further capture the relational features of higher order structures. The higher order adjacency matrix $A_{h}$ is denoted as


(19) \begin{align*}&A_{h}= \begin{bmatrix} H_{lnc} & R_{lnc-mi} & R_{lnc-mr} & R_{lnc-dis} \\ R_{lnc-mi}^{T} & H_{mi} & R_{mi-mr} & R_{mi-dis} \\ R_{lnc-mr}^{T} & R_{mi-mr}^{T} & H_{mr} & R_{mr-dis} \\ R_{lnc-dis}^{T} & R_{mi-dis}^{T} & R_{mr-dis}^{T} & H_{dis} \end{bmatrix}\end{align*}

In the second layer of the high-order GCN, it is assumed that homogeneous nodes within higher order structures exhibit stronger similarity. Based on this assumption, the similarity between nodes is set to the maximum value of 1, rather than using traditional similarity scores in the range of 0 to 1. Accordingly, a new adjacency matrix $A_{h}$ is constructed to more accurately reflect the relational characteristics among nodes in high-order structures, thereby enhancing the model’s expressive power and learning performance. Specifically, the high-order relationship matrix between RNA nodes is defined as $H_{R}(i,j)$, as follows:


(20) \begin{align*}& H_{R}(i,j) = \begin{cases} 1, & \text{ if RNAs}\ i \text{ and}\ j \text{ co-occur in a triangle}, \\ 0, & \text{ otherwise}. \end{cases}\end{align*}

Similarly, the higher order relationship matrix for diseases is expressed as


(21) \begin{align*}& H_{D}(i,j) = \begin{cases} 1, & \text{ if diseases}\ i \text{ and}\ j \text{ co-occur in a triangle}, \\ 0, & \text{ otherwise}. \end{cases}\end{align*}

Conventional GCNs consider only the direct neighborhood relationships between nodes, ignoring the potential associations among closely connected nodes in higher order structures. This limitation may result in insufficient capture of global network information. The multilayer GCN propagation mechanism proposed in this study is defined as follows:


(22) \begin{align*}&H=A_{h}(L\text{eakyRe}LU(A_{e}H^{(0)}W^{(1)}))W^{(2)},\end{align*}

where $A_{e}$ and $A_{h}$ denote the augmented and higher order neighborhood matrices of the heterogeneous network, respectively. Additionally, $W^{(1)}$ and $W^{(2)}$ correspond to trainable weight matrices, while $LeakyReLU$ functions as the nonlinear activation mechanism. The term $H^{(0)}$ represents the randomly initialized embedding matrix. By incorporating high-order structural modeling, the high-order GCN is capable of capturing not only local neighborhood information but also effectively leveraging global associations within the network. This enhances the model’s expressive power and generalization ability. The proposed approach provides a novel tool for in-depth analysis of complex networks.

In this study, the embedding matrix $H$ generated by the high-order GCN is used to obtain the representations of various node types in the ceRNA network, including lncRNAs, miRNAs, and mRNAs. These embeddings are then concatenated to construct a comprehensive representation of the ceRNA network:


(23) \begin{align*}& h_{\text{triplet}}^{\text{concat}} = \left[ h_{\text{lncRNA}_{i}} \mid h_{\text{miRNA}_{j}} \mid h_{\text{mRNA}_{k}} \right]\end{align*}

Subsequently, the spliced representation is subjected to dimensionality reduction or feature transformation by means of a multilayer perceptron:


(24) \begin{align*}&h_{triplet}=MLP(h_{triplet}^{concat})\end{align*}

And the results are weighted and averaged to get the final:


(25) \begin{align*}&h_{triplet}=\alpha_{1}h_{\ln cRNA_{i}}+\alpha_{2}h_{miRNA_{j}}+\alpha_{3}h_{mRNA_{k}}\end{align*}

In the CERDA-HOSR method, the node embedding matrices are computed by multiplying them with the trainable weight matrix $W_{p}$ to compute their connectivity probabilities, which are nonlinearly transformed by a $Sigmoid$ function. For example, the connection probability for the node group $triplet = {lncRNA_{i},miRNA_{j},mRNA_{k}}$ and disease $n$ can be expressed as


(26) \begin{align*}&\hat{y}_{e_{tn}}=Sigmoid ( h_{\mathrm{triplet}}W_{p}h_{n} )\end{align*}

The vectors $h_{{triplet}}$ and $h_{n}$ represent the embedding representations for the node group $triplet$ and node $n$, respectively. Additionally, $W_{p}$ denotes the learnable weight matrix.

Finally, the generated embedding matrix along with the corresponding labels is fed into a binary classification model, such as Logistic Regression or LightGBM, to predict the associations between RNAs and diseases.

### Higher order negative sampling strategy

In the ceRNA association prediction task, the sample imbalance problem and the complex higher order topology of biological networks limit the effectiveness of traditional random negative sampling methods. To ensure that negative samples exhibit structural realism and distributional consistency, this study adopts a high-order structure consistency–based negative sampling strategy to enhance the model’s learning capability.

The proposed negative sampling strategy proceeds as follows. First, a known ceRNA triplet (lncRNA, miRNA, mRNA) is randomly selected from the positive sample set, ensuring that the internal regulatory relationships, i.e. lncRNA–miRNA and miRNA–mRNA are experimentally validated. Then, a disease node that is not associated with this triplet is randomly selected from the complete set of diseases. Subsequently, while keeping the miRNA node fixed, the original lncRNA and mRNA are replaced with other nodes that have established regulatory interactions with the same miRNA, thereby constructing a new triplet-level negative sample. Throughout the sampling process, it is ensured that the generated negative samples do not overlap with any known positive samples. Moreover, the distribution of all node types (lncRNA, mRNA, miRNA, disease) is kept consistent between positive and negative samples to prevent data leakage and enhance model generalizability.

Compared with traditional negative sampling approaches, this strategy maintains the structural integrity of ceRNA triplets and only samples over the disease dimension, ensuring structural consistency. By precomputing candidate disease sets and incorporating a stratified sampling mechanism, the strategy improves sampling efficiency and sample diversity. For fully associated triplets (i.e. those linked to all disease nodes), the method dynamically expands the disease candidate set or adjusts the sampling procedure to prevent failure. This high-order structure-aware sampling design provides high-quality data support for ceRNA network modeling, effectively enhancing the model’s capability to learn ceRNA–disease associations, and offers a more reliable foundation for ceRNA mechanism interpretation and disease prediction. The detailed pseudocode is presented in Algorithm 1.



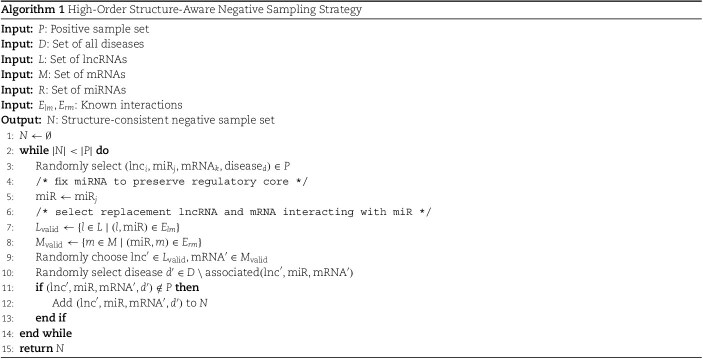



## Experimental results

To evaluate the performance of the model, we adopt AUC-ROC, AUC-PR, Accuracy, F1, Precision, and Recall as evaluation metrics. The classification threshold corresponding to the highest F1 score (0.55) is selected as the final decision boundary for distinguishing positive and negative classes. To evaluate the contribution of each module within the CERDA-HOSR framework, we conducted ablation experiments. In addition, case studies on three representative diseases were performed to demonstrate the practical applicability of CERDA-HOSR in predicting ceRNA–disease associations. Experimental results show that the proposed model achieves more accurate feature representations through high-order structural modeling, thereby validating its effectiveness and generalizability in ceRNA–disease association prediction tasks. The detailed experimental settings are summarized in Table 1.

**Table 1:** Summary of experimental parameter settings

Parameter	Value
Epoch	300
Learning rate	0.001
Layers of GCN	2
Embedding size	64
Train ratio	0.8
Test ratio	0.2
Number of experiments	30
Dropout	0.5
Hidden size	128

In this study, most structural parameters (e.g. the number of GCN layers, embedding dimensions, and hidden layer sizes) and training hyperparameters (e.g. learning rate, dropout rate, and number of training epochs) were determined through a combination of empirical initialization and grid search. Specifically, we adopted the following tuning strategy to evaluate model performance on the dataset and select the optimal parameter configuration: (i) the learning rate was tuned within the range 0.01, 0.005, 0.001 using grid search; (ii) hidden layer size and embedding dimension were evaluated over the set 32, 64, 128; (iii) the dropout rate was tested within the interval [0.3, 0.7].

This parameter search strategy aimed to balance model complexity and generalization ability. The final configuration was selected based on the best performance on the validation set, ensuring the robustness and effectiveness of the model across different datasets.

### Dataset collection and construction

**Figure 3:**
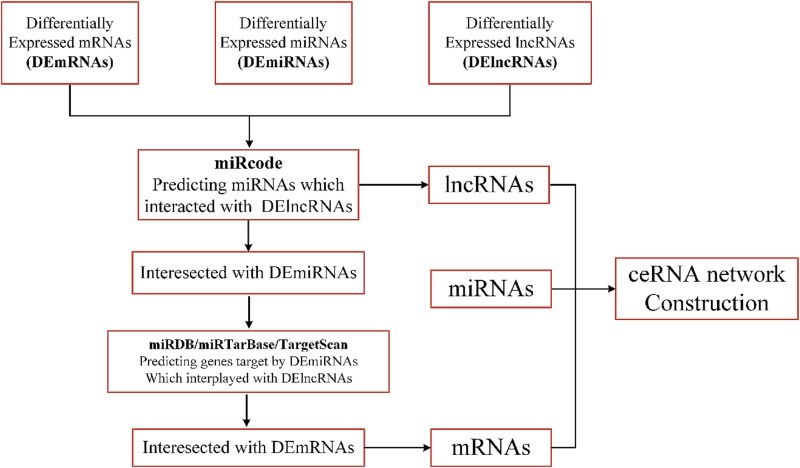
Obtaining differentially expressed lncRNAs, miRNAs, and mRNAs.

The Dataset1 constructed in this study is derived from the authoritative public database LncACTdb 3.0 [47]. This database integrates transcriptomic resources from multiple platforms, including TCGA and GEO, and applies a standardized differential expression analysis pipeline using widely adopted tools such as DESeq2, edgeR, and limma. Under consistent filtering criteria (e.g. $\log _{2}\mathrm{FC}$ > 1 and FDR < 0.05), LncACTdb systematically identified differentially expressed genes from disease-related samples across 537 conditions in 25 species, thereby generating a high-confidence differential expression profile. Figure 3 illustrates the complete pipeline from differential expression analysis to ceRNA network construction.

In addition, LncACTdb 3.0 provides a well-curated set of experimentally validated lncRNA–miRNA–mRNA triplets, manually extracted through literature mining. These interactions form a structurally complete and well-documented ceRNA interaction network. Based on this database, we further filtered for human disease–related data and applied the following data preprocessing steps: removal of duplicate triplets, exclusion of isolated nodes not involved in any known disease association, unification of gene nomenclature based on HGNC standards, and retention of only those lncRNA, miRNA, and mRNA nodes that co-occur in at least one triplet to ensure network connectivity and expression consistency.

As a result, the final Dataset1 comprises 218 lncRNAs, 605 miRNAs, 1051 mRNAs, and 314 human diseases, encompassing a total of 2115 ceRNA network–disease regulatory associations. This dataset offers broad coverage of molecular regulatory mechanisms under various complex disease contexts and provides a robust foundation for subsequent modeling and disease prediction tasks.

In addition, multiple publicly available datasets were utilized to perform RNA–disease association prediction experiments [48]. The detailed properties of each dataset are summarized in Table 2.

**Table 2:** Dataset contents

Datasets	RNAs	Diseases	Edges
Circ2D v1.0	585	88	6650
Circ2D v2.0	3077	312	4201
HMDD v2.0	495	383	5430
HMDD v3.0	1102	850	32 226
HMDD v4.0	1896	2360	53 530
Dataset1	1936	314	5157

### Comparison with baseline

For simplicity of presentation, the proposed model CERDA-HOSR is hereafter referred to as **CERDA**. To evaluate the performance of CERDA in RNA–disease association prediction, we conducted comparative experiments against several baseline methods across multiple datasets. The experimental results are presented in Table 3. Detailed descriptions of the baseline methods are provided below.

**Table 3:** Comparison results of different methods (**Bold** highlights the best results)

**Dataset**	**Methods**	**AUC**	**AUPR**	**ACC**	**Pre**	**Recall**	**F1**
CircR2 Disease v1.0	VGAMF	0.720$\pm $0.015	0.728$\pm $0.018	0.667$\pm $0.020	0.676$\pm $0.012	0.66$\pm $0.009	0.668$\pm $0.017
	NRMFLDA	0.650$\pm $0.022	0.589$\pm $0.012	0.611$\pm $0.014	0.574$\pm $0.018	0.628$\pm $0.023	0.601$\pm $0.022
	VGAE-MDA	0.923$\pm $0.005	0.942$\pm $0.013	0.828$\pm $0.005	0.840$\pm $0.016	0.868$\pm $0.009	0.854$\pm $0.007
	MAMFGET	**0.978$\pm $0.017**	**0.976$\pm $0.013**	**0.961$\pm $0.017**	**0.974$\pm $0.025**	0.947$\pm $0.016	**0.960$\pm $0.019**
	MAGCN	0.734$\pm $0.016	0.753$\pm $0.009	0.654$\pm $0.011	0.803$\pm $0.016	0.417$\pm $0.019	0.541$\pm $0.014
	CERDA	0.921$\pm $0.006	0.932$\pm $0.007	0.951$\pm $0.005	0.946$\pm $0.012	**0.958$\pm $0.007**	0.952$\pm $0.006
CircR2 Disease v2.0	VGAMF	0.821$\pm $0.012	0.832$\pm $0.011	0.816$\pm $0.009	0.856$\pm $0.012	0.814$\pm $0.007	0.835$\pm $0.006
	NRMFLDA	0.886$\pm $0.009	**0.902$\pm $0.007**	0.894$\pm $0.006	**0.917$\pm $0.012**	0.875$\pm $0.005	0.892$\pm $0.007
	VGAE-MDA	0.872$\pm $0.011	0.863$\pm $0.012	0.891$\pm $0.007	0.889$\pm $0.016	0.835$\pm $0.007	0.862$\pm $0.010
	MAMFGET	**0.925$\pm $0.021**	0.895$\pm $0.034	0.865$\pm $0.022	0.817$\pm $0.034	**0.942$\pm $0.018**	0.874$\pm $0.023
	MAGCN	0.849$\pm $0.015	0.841$\pm $0.016	0.767$\pm $0.018	0.833$\pm $0.014	0.669$\pm $0.019	0.742$\pm $0.012
	CERDA	0.912$\pm $0.017	0.901$\pm $0.018	**0.926$\pm $0.016**	0.873$\pm $0.019	0.917$\pm $0.029	**0.895$\pm $0.023**
HMDD V2.0	VGAMF	0.931$\pm $0.005	0.923$\pm $0.008	0.826$\pm $0.005	0.835$\pm $0.019	0.847$\pm $0.011	0.841$\pm $0.016
	NRMFLDA	0.863$\pm $0.008	0.893$\pm $0.004	0.882$\pm $0.003	0.864$\pm $0.012	0.887$\pm $0.004	0.876$\pm $0.007
	VGAE-MDA	0.892$\pm $0.011	0.911$\pm $0.006	**0.886$\pm $0.009**	**0.915$\pm $0.019**	0.857$\pm $0.011	**0.889$\pm $0.015**
	MAMFGET	**0.949$\pm $0.003**	**0.941$\pm $0.005**	0.880$\pm $0.004	0.849$\pm $0.019	**0.924$\pm $0.011**	0.885$\pm $0.005
	MAGCN	0.848$\pm $0.015	0.844$\pm $0.012	0.774$\pm $0.014	0.833$\pm $0.015	0.683$\pm $0.019	0.751$\pm $0.016
	CERDA	0.912$\pm $0.007	0.896$\pm $0.008	0.863$\pm $0.011	0.818$\pm $0.006	0.874$\pm $0.013	0.846$\pm $0.003
HMDD V3.0	VGAMF	0.846$\pm $0.008	0.830$\pm $0.007	0.823$\pm $0.008	0.831$\pm $0.016	0.823$\pm $0.008	0.829$\pm $0.009
	NRMFLDA	0.863$\pm $0.009	0.872$\pm $0.003	0.863$\pm $0.008	0.872$\pm $0.019	0.863$\pm $0.014	0.883$\pm $0.004
	VGAE-MDA	0.892$\pm $0.007	0.903$\pm $0.006	**0.911$\pm $0.005**	0.903$\pm $0.017	**0.911$\pm $0.010**	0.904$\pm $0.007
	MAMFGET	**0.960$\pm $0.002**	**0.956$\pm $0.004**	0.890$\pm $0.008	0.862$\pm $0.025	0.930$\pm $0.021	0.894$\pm $0.008
	MAGCN	0.856$\pm $0.023	0.858$\pm $0.015	0.793$\pm $0.014	0.836$\pm $0.019	0.728$\pm $0.008	0.779$\pm $0.009
	CERDA	0.921$\pm $0.006	0.916$\pm $0.007	0.906$\pm $0.007	**0.916$\pm $0.016**	0.906$\pm $0.008	**0.906$\pm $0.007**
HMDD V4.0	VGAMF	0.865$\pm $0.008	0.895$\pm $0.009	0.902$\pm $0.009	0.873$\pm $0.012	0.861$\pm $0.006	0.867$\pm $0.010
	NRMFLDA	0.884$\pm $0.009	0.896$\pm $0.008	0.904$\pm $0.009	0.942$\pm $0.011	0.891$\pm $0.013	0.916$\pm $0.008
	VGAE-MDA	0.936$\pm $0.006	0.940$\pm $0.006	0.916$\pm $0.007	0.918$\pm $0.008	**0.954$\pm $0.013**	0.936$\pm $0.007
	MAMFGET	0.950$\pm $0.003	0.946$\pm $0.004	0.877$\pm $0.004	0.859$\pm $0.009	0.902$\pm $0.018	0.879$\pm $0.013
	MAGCN	0.949$\pm $0.014	**0.954$\pm $0.018**	0.871$\pm $0.016	0.941$\pm $0.018	0.790$\pm $0.019	0.859$\pm $0.011
	CERDA	**0.952$\pm $0.005**	0.943$\pm $0.005	**0.934$\pm $0.006**	**0.943$\pm $0.019**	0.941$\pm $0.008	**0.942$\pm $0.007**
Dataset1	VGAMF	0.850$\pm $0.006	0.914$\pm $0.007	0.904$\pm $0.005	0.909$\pm $0.014	0.879$\pm $0.011	0.894$\pm $0.006
	NRMFLDA	0.850$\pm $0.005	0.919$\pm $0.006	0.903$\pm $0.006	0.895$\pm $0.012	0.891$\pm $0.009	0.893$\pm $0.003
	VGAE-MDA	0.827$\pm $0.007	0.829$\pm $0.005	0.775$\pm $0.006	0.763$\pm $0.013	0.741$\pm $0.008	0.752$\pm $0.007
	MAMFGET	0.852$\pm $0.013	0.861$\pm $0.006	0.832$\pm $0.022	0.863$\pm $0.016	0.852$\pm $0.012	0.812$\pm $0.013
	MAGCN	0.872$\pm $0.012	0.881$\pm $0.014	0.842$\pm $0.011	0.882$\pm $0.019	0.876$\pm $0.008	0.873$\pm $0.009
	CERDA	**0.936$\pm $0.005**	**0.945$\pm $0.004**	**0.946$\pm $0.005**	**0.961$\pm $0.016**	**0.941$\pm $0.007**	**0.956$\pm $0.006**

VGAMF [21]: This model predicts miRNA–disease associations by integrating nonlinear and linear representations within fully connected neural networks.

NRMFLDA [49]: A method for predicting lncRNA–disease associations that integrates matrix factorization with disease neighborhood regularization to effectively infer disease-related lncRNAs.

VGAE-MDA [29]: This method fuses the training outputs from two sub-networks to derive the final prediction.

MAMFGET [50]: MAMFGET extends GATs with adaptive multimodal fusion and contrastive learning, enabling the integration of complementary information from multiple networks and alleviating over-smoothing. This design yields more robust miRNA–disease feature representations.

MAGCN [14]: This method identifies novel biomarkers by leveraging known miRNA–disease associations and LMIs, using LMIs as a bridge to reduce data sparsity and bias compared with similarity-based approaches.

Experimental results demonstrate that CERDA achieves superior performance across all evaluation metrics, highlighting its effectiveness in RNA–disease association prediction tasks. Compared with other methods, CERDA is able to capture complex feature associations in ceRNA networks more accurately by virtue of the higher order GCN and attention mechanism, and has obvious advantages in datasets of different sizes and complexities, proving its wide applicability in a variety of bioinformatics tasks. In particular, CERDA achieves the highest prediction performance in the self-constructed Dataset1 dataset, further validating its applicability in large-scale complex network data. The experimental results fully demonstrate the reliability and validity of CERDA in RNA-disease association prediction, providing strong support for the in-depth study of ceRNA networks.

In addition to the influence of high-order topological structures, this study further investigates the network topology characteristics of the selected datasets. Three metrics are employed to evaluate the topological properties: Average Eigenvector Centrality (AEC), Average Closeness Centrality (ACC), and Average Degree Centrality (ADC). Table 4 summarizes the network topology attributes for each dataset.

**Table 4:** Network topology attributes (**Bold** indicates best result)

Datasets	AEC	ACC	ADC
Circ2D v1.0	0.0137	0.0728	0.0029
Circ2D v2.0	0.0068	0.1444	0.0007
HMDD V2.0	**0.0198**	**0.3222**	**0.0141**
HMDD V3.0	0.0124	0.3168	0.0093
HMDD V4.0	0.0066	0.2904	0.0034
Datasets1	0.0110	0.2270	0.0022

Overall, the higher order topological characteristics of the network are the key factors affecting the model performance. The HMDD series datasets, especially V2.0 and V3.0, provide a good foundation for the model performance by virtue of their powerful higher order topological characteristics.

### Validation results and significance analysis

**Figure 4:**
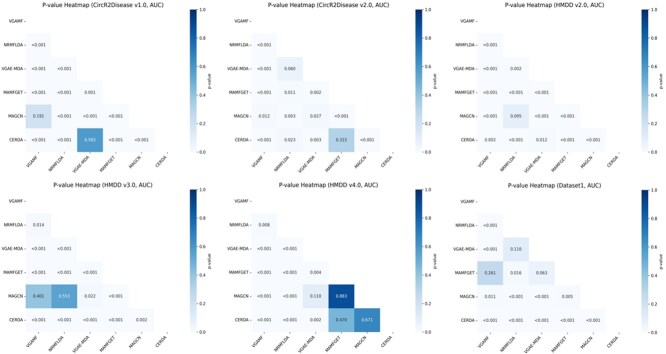
Statistical significance analysis of models on different datasets.

To verify whether the observed performance improvements of CERDA were statistically significant, we conducted pairwise comparisons using Welch’s t-test based on the AUC-ROC scores across all datasets. The resulting $P$-values are visualized as heatmaps in Fig. 4, where darker shades indicate stronger statistical significance. In most cases, the differences between CERDA and other baseline methods are statistically significant ($P$ < 0.05), thereby supporting our claims regarding the model’s superior performance. While CERDA generally demonstrates consistent and robust advantages across diverse datasets, the relative benefit may vary depending on specific data characteristics. Overall, these results further validate the effectiveness and generalizability of CERDA in RNA–disease association prediction.

As shown in Table 3 and Table 5, the experimental results under different validation strategies indicate that conventional $k$-fold cross-validation tends to overestimate the generalization performance of models on unseen diseases. In contrast, disease-stratified cross-validation, by ensuring that the disease categories in the training and test sets are completely nonoverlapping, more closely reflects a realistic “cold-start” prediction scenario. Under this setting, the model must make predictions for diseases that have never been observed during training, and thus cannot rely on feature patterns of previously known diseases, which substantially increases the task difficulty.

**Table 5:** Comparison results of five-fold cross-validation stratified by disease (**Bold** highlights the best results)

**Dataset**	**Methods**	**AUC**	**AUPR**	**ACC**	**Pre**	**Recall**	**F1**
CircR2 Disease v1.0	VGAMF	0.420$\pm $0.015	0.464$\pm $0.018	0.504$\pm $0.020	0.576$\pm $0.013	0.564$\pm $0.011	0.570$\pm $0.009
	NRMF-LDA	0.462$\pm $0.012	0.459$\pm $0.012	0.512$\pm $0.014	0.574$\pm $0.021	0.628$\pm $0.016	0.599$\pm $0.122
	VGAE-MDA	0.523$\pm $0.017	**0.562$\pm $0.023**	0.493$\pm $0.015	0.486$\pm $0.024	0.554$\pm $0.017	0.518$\pm $0.016
	MAMFGET	0.393$\pm $0.164	0.456$\pm $0.102	**0.585$\pm $0.081**	0.608$\pm $0.119	**0.748$\pm $0.398**	0.583$\pm $0.253
	MAGCN	0.413$\pm $0.102	0.438$\pm $0.052	0.481$\pm $0.039	0.310$\pm $0.242	0.030$\pm $0.025	0.051$\pm $0.040
	CERDA	**0.552$\pm $0.124**	0.532$\pm $0.017	0.515$\pm $0.016	**0.646$\pm $0.020**	0.585$\pm $0.019	**0.614$\pm $0.014**
CircR2 Disease v2.0	VGAMF	0.412$\pm $0.016	0.432$\pm $0.011	0.462$\pm $0.019	0.556$\pm $0.012	0.441$\pm $0.013	0.492$\pm $0.009
	NRMF-LDA	0.465$\pm $0.019	0.502$\pm $0.017	0.449$\pm $0.026	0.471$\pm $0.031	0.475$\pm $0.206	0.473$\pm $0.103
	VGAE-MDA	0.427$\pm $0.011	0.436$\pm $0.012	0.419$\pm $0.021	0.498$\pm $0.032	0.468$\pm $0.241	0.483$\pm $0.129
	MAMFGET	**0.583$\pm $0.032**	**0.554$\pm $0.057**	**0.616$\pm $0.027**	0.572$\pm $0.054	**0.859$\pm $0.099**	**0.683$\pm $0.043**
	MAGCN	0.457$\pm $0.041	0.470$\pm $0.029	0.501$\pm $0.002	0.171$\pm $0.383	0.002$\pm $0.005	0.005$\pm $0.010
	CERDA	0.512$\pm $0.017	0.532$\pm $0.018	0.562$\pm $0.021	**0.573$\pm $0.021**	0.617$\pm $0.023	0.594$\pm $0.016
HMDD v2.0	VGAMF	0.650$\pm $0.011	0.643$\pm $0.019	0.613$\pm $0.014	0.641$\pm $0.018	0.631$\pm $0.044	0.636$\pm $0.024
	NRMF-LDA	0.643$\pm $0.049	0.632$\pm $0.027	0.627$\pm $0.059	0.592$\pm $0.044	0.624$\pm $0.019	0.608$\pm $0.025
	VGAE-MDA	0.712$\pm $0.019	0.706$\pm $0.034	0.742$\pm $0.039	0.762$\pm $0.057	0.772$\pm $0.061	0.767$\pm $0.042
	MAMFGET	**0.881$\pm $0.016**	**0.889$\pm $0.018**	0.812$\pm $0.020	0.852$\pm $0.054	0.751$\pm $0.037	0.796$\pm $0.011
	MAGCN	0.793$\pm $0.021	0.777$\pm $0.025	0.537$\pm $0.012	**0.896$\pm $0.048**	0.084$\pm $0.030	0.153$\pm $0.049
	CERDA	0.811$\pm $0.029	0.825$\pm $0.041	**0.831$\pm $0.016**	0.792$\pm $0.019	**0.815$\pm $0.031**	**0.803$\pm $0.018**
HMDD v3.0	VGAMF	0.812$\pm $0.016	0.793$\pm $0.035	0.826$\pm $0.029	0.806$\pm $0.019	0.801$\pm $0.016	0.803$\pm $0.012
	NRMF-LDA	0.812$\pm $0.019	0.821$\pm $0.028	0.824$\pm $0.036	0.826$\pm $0.054	0.831$\pm $0.049	0.828$\pm $0.036
	VGAE-MDA	0.851$\pm $0.018	0.846$\pm $0.019	0.863$\pm $0.026	0.843$\pm $0.029	0.863$\pm $0.039	0.853$\pm $0.024
	MAMFGET	**0.922$\pm $0.014**	**0.925$\pm $0.020**	0.851$\pm $0.019	0.865$\pm $0.038	0.826$\pm $0.047	0.844$\pm $0.024
	MAGCN	0.829$\pm $0.019	0.810$\pm $0.020	0.528$\pm $0.010	**0.938$\pm $0.024**	0.059$\pm $0.021	0.111$\pm $0.036
	CERDA	0.852$\pm $0.014	0.863$\pm $0.019	**0.873$\pm $0.049**	0.862$\pm $0.048	**0.884$\pm $0.059**	**0.873$\pm $0.038**
HMDD v4.0	VGAMF	0.813$\pm $0.014	0.823$\pm $0.023	0.835$\pm $0.049	0.826$\pm $0.035	0.841$\pm $0.032	0.833$\pm $0.024
	NRMF-LDA	0.825$\pm $0.019	0.813$\pm $0.044	0.834$\pm $0.034	0.852$\pm $0.043	0.824$\pm $0.048	0.838$\pm $0.032
	VGAE-MDA	0.872$\pm $0.015	**0.881$\pm $0.029**	0.869$\pm $0.031	0.874$\pm $0.042	**0.882$\pm $0.043**	**0.878$\pm $0.030**
	MAMFGET	0.837$\pm $0.013	0.829$\pm $0.011	0.776$\pm $0.011	0.789$\pm $0.016	0.752$\pm $0.020	0.770$\pm $0.011
	MAGCN	**0.874$\pm $0.009**	0.871$\pm $0.008	0.650$\pm $0.022	**0.927$\pm $0.010**	0.327$\pm $0.051	0.481$\pm $0.054
	CERDA	0.852$\pm $0.016	0.846$\pm $0.061	**0.893$\pm $0.046**	0.849$\pm $0.068	0.865$\pm $0.049	0.857$\pm $0.042
Dataset 1	VGAMF	0.823$\pm $0.015	0.831$\pm $0.029	0.812$\pm $0.049	0.835$\pm $0.056	0.834$\pm $0.026	0.834$\pm $0.031
	NRMF-LDA	0.752$\pm $0.019	0.783$\pm $0.023	0.794$\pm $0.029	0.813$\pm $0.035	0.824$\pm $0.046	0.818$\pm $0.029
	VGAE-MDA	0.746$\pm $0.016	0.723$\pm $0.032	0.738$\pm $0.049	0.735$\pm $0.048	0.768$\pm $0.058	0.751$\pm $0.037
	MAMFGET	0.824$\pm $0.016	0.843$\pm $0.023	0.813$\pm $0.019	0.823$\pm $0.039	0.834$\pm $0.036	0.828$\pm $0.027
	MAGCN	0.783$\pm $0.046	0.793$\pm $0.039	0.784$\pm $0.049	0.812$\pm $0.068	0.812$\pm $0.047	0.812$\pm $0.041
	CERDA	**0.856$\pm $0.016**	**0.863$\pm $0.049**	**0.853$\pm $0.035**	**0.873$\pm $0.038**	**0.861$\pm $0.049**	**0.867$\pm $0.031**

**Figure 5:**
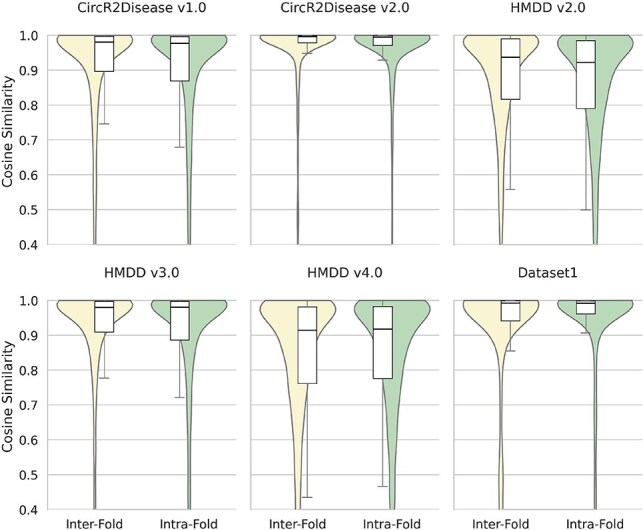
The distribution of disease similarity for Intra-Fold and Inter-Fold across different datasets.

Figure 5 presents the distributional comparison of intra-fold similarity (green) and inter-fold similarity (yellow) across different datasets. The $x$-axis denotes the two types of similarity, while the $y$-axis shows the cosine similarity scores. Intra-fold similarity refers to the similarity among samples within the same fold, which is typically higher since samples in the same fold originate from the same dataset and are often interrelated. In contrast, inter-fold similarity measures the similarity between samples from different folds, which is generally lower because training and testing sets do not share disease categories. These quantitative analyses strongly support the effectiveness of the stratified cross-validation strategy: not only does it preserve class balance in terms of sample numbers, but it also maintains intra-cluster consistency within diseases and distributional stability across folds, thereby ensuring the reliability of model evaluation and the generalizability of the results.

Compared with ordinary cross-validation, the disease-stratified strategy generally leads to decreases in classification metrics such as AUC, AUPR, and ACC across most datasets and models. However, this change is expected and reflects the true performance level of the models under more stringent evaluation conditions. Furthermore, the use of five-fold disease-stratified validation exhibits slightly higher mean values and smaller standard deviations on some datasets, suggesting that increasing the number of folds helps mitigate fluctuations caused by sample distribution differences and improves evaluation stability.

Overall, disease-stratified cross-validation not only reveals the true performance of models in cold-start scenarios, but also provides a more reliable reference for subsequent biological validation.

### Ablation experiment

To validate that the performance improvements of CERDA stem from its higher order structural design rather than general architectural choices, we conduct ablation experiments by systematically removing three key components: the high-order GCN (HoGCN), high-order GAT (HoGAT), and high-order negative sampling strategy (HoNS). As shown in Table 6, CERDA significantly outperforms standard GCN and GAT models. Furthermore, the removal of each higher order module leads to a consistent performance drop across all evaluation metrics, demonstrating the effectiveness and necessity of incorporating higher order structures into the model.

**Table 6:** Ablation experiments (**Bold** highlights the best results)

**Dataset**	**Method**	**AUC-ROC**	**AUC-PR**	**ACC**	**F1**
Circ2Disease v1.0	CERDA	**0.921**	**0.932**	**0.951**	**0.952**
	GCN	0.592	0.612	0.546	0.516
	GAT	0.540	0.530	0.510	0.543
	CERDA-GCN	0.885	0.873	0.812	0.823
	CERDA-GAT	0.913	0.915	0.822	0.837
	w/o HoNS	0.910	0.918	0.847	0.858
Circ2Disease v2.0	CERDA	**0.912**	**0.901**	**0.926**	**0.895**
	GCN	0.562	0.573	0.616	0.576
	GAT	0.543	0.532	0.527	0.534
	CERDA-GCN	0.812	0.816	0.806	0.821
	CERDA-GAT	0.822	0.842	0.831	0.870
	w/o HoNS	0.876	0.896	0.882	0.871
HMDD V2.0	CERDA	0.912	0.896	0.863	0.846
	GCN	0.622	0.612	0.646	0.597
	GAT	0.587	0.530	0.576	0.586
	CERDA-GCN	0.859	0.861	0.819	0.817
	CERDA-GAT	**0.901**	**0.933**	**0.921**	**0.920**
	w/o HoNS	0.934	0.957	0.878	0.872
HMDD V3.0	CERDA	**0.921**	**0.916**	**0.906**	**0.924**
	GCN	0.692	0.612	0.646	0.656
	GAT	0.640	0.642	0.615	0.637
	CERDA-GCN	0.786	0.792	0.805	0.799
	CERDA-GAT	0.826	0.815	0.829	0.817
	w/o HoNS	0.863	0.846	0.834	0.829
HMDD V4.0	CERDA	**0.922**	**0.933**	**0.934**	**0.942**
	GCN	0.552	0.526	0.546	0.516
	GAT	0.613	0.643	0.627	0.634
	CERDA-GCN	0.912	0.893	0.876	0.907
	CERDA-GAT	0.836	0.828	0.843	0.836
	w/o HoNS	0.884	0.860	0.846	0.863
Dataset1	CERDA	**0.956**	**0.965**	**0.946**	**0.956**
	GCN	0.522	0.517	0.546	0.583
	GAT	0.545	0.539	0.562	0.573
	CERDA-GCN	0.864	0.872	0.843	0.862
	CERDA-GAT	0.882	0.873	0.893	0.871
	w/o HoNS	0.902	0.893	0.904	0.873

Compared with other datasets, CERDA-HOSR demonstrates a more significant performance advantage on Dataset1. We attribute this to several key factors:

(i) Denser and more balanced network structure: Although Dataset1 contains a relatively smaller number of RNA–disease associations, its nodes are more evenly distributed across different RNA types and diseases. This facilitates more effective message passing in GCN layers, enhancing representation learning.
(ii) Greater node-type heterogeneity: Dataset1 incorporates three types of RNA molecules—circRNA, lncRNA, and miRNA, resulting in a more complex and heterogeneous topological structure. The high-order structure representation in CERDA-HOSR is particularly well-suited to such heterogeneous graphs, capturing multi-hop and multi-type regulatory patterns.
(iii) High-quality literature-validated data sources: Unlike datasets relying on similarity inference or automated database generation, most RNA–disease associations in Dataset1 are curated from experimental literature, which reduces label noise and improves biological consistency, thereby supporting stable and generalizable learning.
  Overall, CERDA performs exceptionally well on datasets rich in high-order structural features (e.g. the HMDD series and Dataset1), confirming its advantage in modeling complex graph structures. In contrast, in datasets with relatively weaker high-order structures, such as CircR2Disease, the contribution of HoGCN is more pronounced, while other modules have a marginal impact. These results suggest that CERDA can flexibly adapt its module effectiveness based on the structural characteristics of the dataset, achieving strong performance across diverse settings.

### Case studies

**Figure 6:**
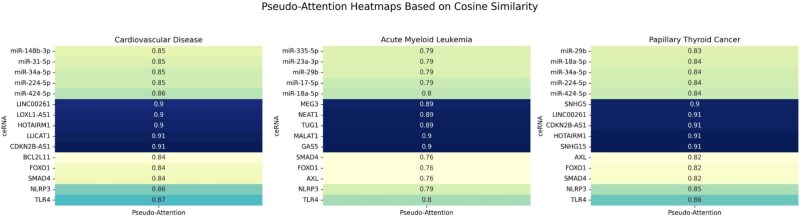
The top-5 attention nodes of the three diseases.

**Figure 7:**
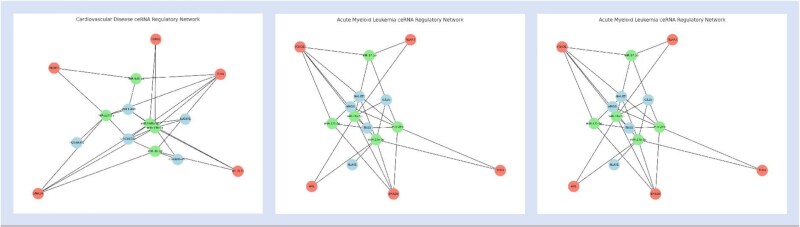
Visualization of the top-20 ceRNA network for three diseases.

Cardiovascular disease, acute myeloid leukemia (AML), and papillary thyroid carcinoma are three major diseases that pose serious threats to human health. Cardiovascular disease remains the leading cause of death worldwide; AML is characterized by rapid progression and poor treatment outcomes; and papillary thyroid carcinoma, though less aggressive, requires timely diagnosis and intervention to improve prognosis. To evaluate the practical applicability of the proposed CERDA-HOSR model in predicting ceRNA–disease associations, we conducted case studies on these three representative diseases. The corresponding prediction results are presented in Table 7, Table 8, and Table 9. Figure 6 and Fig. 7, respectively, depict the top-5 attention-weighted nodes and the top-20 predicted ceRNA networks for each of the three diseases.

**Table 7:** Case study on cardiovascular diseases (**Bold** indicates literature-confirmed subpaths)

**Disease**	**LncRNA**	**miRNA**	**mRNA**	**Evidence (PMID)**
**Cardiovascular diseases**	LINC00261	miR-148b-3p	SMAD4	34239606 / 39690389 / 40314862
	LOXL1-AS1	miR-148b-3p	TLR4	34633594 / 38936497 / 40171680
	LUCAT1	miR-148b-3p	BCL2L11	39741278 / 38936497 / 39689089
	LOXL1-AS1	**miR-424-5p**	**TLR4**	34633594 / 33985567 / 32932194 (35667541)
	LOXL1-AS1	**miR-424-5p**	**NLRP3**	34633594 / 33985567 / 34906598 (39996771)
	CDKN2B-AS1	miR-34a-5p	BCL2L11	33165136 / 37806095 / 39689089
	LOXL1-AS1	**miR-224-5p**	**SMAD4**	34633594 / 39421730 / 38182899 (34408438)
	LOXL1-AS1	miR-34a-5p	BCL2L11	32453072 / 35879917 / 37772397
	LINC00261	**miR-34a-5p**	**SMAD4**	32558131 / 37806095 / 38182899 (32929850)
	LINC00261	**miR-224-5p**	**TLR4**	34239606 / 39421730 / 32932194 (34092750)
	LUCAT1	miR-148b-3p	TLR4	37197923 / 27505319 / 32932194
	CDKN2B-AS1	miR-34a-5p	TLR4	32385877 / 33115945 / 32932194
	LINC00261	**miR-148b-3p**	**FOXO1**	34239606 / 37806095 / 31765819 (39602888)
	LINC00261	**miR-31-5p**	**BCL2L11**	34239606 / 32428930 / 37772397 (31497013)
	**LUCAT1**	**miR-34a-5p**	FOXO1	37197923 / 33115945 / 31765819 (38757341)
	CDKN2B-AS1	miR-31-5p	TLR4	32385877 / 32428930 / 32932194
	LOXL1-AS1	**miR-224-5p**	**NLRP3**	32453072 / 39421730 / 34906598 (37092127)
	CDKN2B-AS1	**miR-31-5p**	**SMAD4**	32385877 / 32428930 / 38182899 (35406680)
	HOTAIRM1	**miR-224-5p**	**SMAD4**	40155530 / 39421730 / 38182899 (34528447)
	CDKN2B-AS1	**miR-34a-5p**	**FOXO1**	32385877 / 33115945 / 31765819 (37506143)

**Table 8:** Case study on acute myeloid leukemia (**Bold** indicates literature-confirmed subpaths)

**Disease**	**LncRNA**	**miRNA**	**mRNA**	**Evidence (PMID)**
**Acute myeloid leukemia**	**MALAT1**	**miR-18a-5p**	AXL	34394903 / 31523391 / 28516360 (39881079)
	GAS5	**miR-17-5p**	**FOXO1**	36061350 / 33241756 / 38211590 (24810926)
	**MALAT1**	**miR-23a-3p**	**SMAD4**	34394903 / 30246348 / 11244507 (39118664, 32722415)
	**TUG1**	**miR-335-5p**	SMAD4	30551433 / 37715395 / 11244507 (28205334)
	**MALAT1**	**miR-18a-5p**	**FOXO1**	38211590 / 31523391 / 38211590 (39881079,24810926)
	**GAS5**	**miR-23a-3p**	**TLR4**	36061350 / 30246348 / 36183949 (30389135,36916084)
	**MALAT1**	**miR-23a-3p**	FOXO1	34394903 / 30246348 / 38211590 (40395909)
	**TUG1**	**miR-335-5p**	FOXO1	30551433 / 37715395 / 38211590 (32341656)
	NEAT1	miR-23a-3p	AXL	32179410 / 30246348 / 28516360
	**MALAT1**	**miR-17-5p**	**NLRP3**	38211590 / 33241756 / 34211462 (31257497, 36341225)
	**MEG3**	**miR-29b**	**FOXO1**	37702691 / 28611288 / 38211590 (27035110, 36652981)
	**GAS5**	**miR-29b**	SMAD4	36061350 / 28611288 / 11244507 (38132140)
	**MALAT1**	**miR-335-5p**	SMAD4	38211590 / 37715395 / 11244507 (35008626)
	**MEG3**	**miR-29b**	TLR4	37702691 / 28611288 / 36183949 (27035110, 33235469)
	**MEG3**	**miR-18a-5p**	NLRP3	37702691 / 31523391 / 34211462 (39881079)
	TUG1	miR-18a-5p	NLRP3	30551433 / 31523391 / 34211462
	**MALAT1**	**miR-23a-3p**	AXL	38211590 / 30246348 / 28516360 (40395909)
	**TUG1**	**miR-29b**	TLR4	30551433 / 28611288 / 36183949 (34540908)
	**GAS5**	**miR-18a-5p**	**SMAD4**	36061350 / 36061350 / 11244507 (34526965, 37724150)
	**GAS5**	**miR-18a-5p**	NLRP3	36061350 / 36061350 / 34211462 (34526965)

**Table 9:** Case study on papillary thyroid cancer (**Bold** indicates literature-confirmed subpaths)

**Disease**	**LncRNA**	**miRNA**	**mRNA**	**Evidence (PMID)**
**Papillary thyroid cancer**	SNHG5	**miR-34a-5p**	**AXL**	35338124 / 33706708 / 36203174 (38897346)
	**HOTAIRM1**	**miR-34a-5p**	**AXL**	32951513 / 33706708 / 36203174 (36289596,38897346)
	SNHG5	**miR-224-5p**	**TLR4**	35338124 / 37410095 / 31894666 (34092750)
	SNHG15	**miR-34a-5p**	**SMAD4**	30237435 / 33706708 / 35462659 (36482069)
	SNHG5	**miR-18a-5p**	**FOXO1**	35338124 / 25124853 / 35865479 (31534842)
	**CDKN2B-AS1**	**miR-424-5p**	**SMAD4**	35750242 / 34800440 / 35462659 (32801906, 34868211)
	LINC00261	miR-18a-5p	AXL	39552712 / 25124853 / 36203174
	**HOTAIRM1**	**miR-29b**	**TLR4**	32951513 / 35530352 / 31894666 (35711826, 33523607)
	**HOTAIRM1**	**miR-29b**	**AXL**	32951513 / 35530352 / 36203174 (35711826, 21731696)
	CDKN2B-AS1	miR-224-5p	AXL	35750242 / 37410095 / 36203174
	**SNHG5**	**miR-29b**	**FOXO1**	35338124 / 35530352 / 35865479 (34298743, 38646331)
	LINC00261	**miR-29b**	**SMAD4**	39552712 / 35530352 / 35865479 (24444023)
	HOTAIRM1	**miR-424-5p**	**NLRP3**	32951513 / 34800440 / – (38691277)
	CDKN2B-AS1	**miR-34a-5p**	**TLR4**	35750242 / 533706708 / 31894666 (38824834)
	HOTAIRM1	**miR-18a-5p**	**TLR4**	32951513 / 25124853 / 31894666 (32815784)
	SNHG15	**miR-424-5p**	**SMAD4**	30237435 / 34800440 / 35865479 (28714374)
	HOTAIRM1	**miR-424-5p**	**TLR4**	32951513 / 34800440 / 31894666 (30115538)
	**CDKN2B-AS1**	**miR-424-5p**	**AXL**	35750242 / 34800440 / 36203174 (33820740, 34542165)
	LINC00261	**miR-29b**	**NLRP3**	39552712 / 35530352 / – (32160394)
	CDKN2B-AS1	**miR-18a-5p**	**SMAD4**	35750242 / 25124853 / 35865479 (31257489)

In these tables, several predicted RNA–disease associations are supported by literature evidence (as indicated by PMIDs), while those without citation may reflect relationships not yet experimentally validated due to limited research, but which may still be biologically relevant. The majority of predicted ceRNA regulatory networks have received cross-validation from existing studies, suggesting that the identified RNA molecules play regulatory roles in the pathogenesis and progression of these diseases. This supports the reliability and biological significance of the model’s predictions and provides a solid foundation for further mechanistic studies and potential clinical applications.

Notably, although some predicted ceRNA triplets are not yet recorded as complete regulatory modules in current databases, all constituent nodes (e.g. miR-148b-3p, SMAD4, LOXL1-AS1) have been individually reported as closely associated with cardiovascular disease in multiple studies. This indicates strong biological plausibility and suggests that the predicted triplets may reflect previously uncharacterized but genuine regulatory pathways, offering valuable guidance for future experimental validation.

Furthermore, we found that several subpaths within the predicted triplets—such as miR-148b-3p $\rightarrow $ SMAD4 and LOXL1-AS1 $\rightarrow $ miR-424-5p have been partially supported across different databases, providing additional corroboration of the model’s predictions. These findings enhance the interpretability of CERDA-HOSR and highlight its potential to inform RNA-based therapeutic strategies, including RNA interference (RNAi), antisense oligonucleotides (ASO), and small-molecule drug development.

## Discussion and conclusion

In this study, we proposed CERDA, a ceRNA–disease association prediction model that integrates high-order structural information into graph representation learning. Experimental results demonstrate that CERDA achieves consistently superior performance compared with baseline methods, particularly on datasets with rich high-order topology. Ablation studies confirm that the incorporation of high-order features significantly enhances model accuracy and robustness, enabling more effective modeling of complex, heterogeneous biological networks.

Nevertheless, CERDA has several limitations: (i) its representational power diminishes on datasets lacking high-order structural information; (ii) the model may underperform under imbalanced RNA–disease data distributions; and (iii) the increased computational cost of high-order mechanisms may limit scalability to large datasets. Although the proposed high-order attention design represents an incremental enhancement over conventional GCN and GAT models, we believe this modification has substantial practical value. Systematic comparisons and statistical analyses demonstrate that CERDA more effectively captures cross-type, multi-hop dependencies, especially in sparse or structurally complex biological graphs.

Future work will focus on improving CERDA’s performance on low-structure datasets, enhancing its adaptability to imbalanced data, and optimizing its computational efficiency to support large-scale deployment. Particular attention will also be devoted to addressing the ”cold-start” problem, for instance by designing more effective strategies to handle previously unseen diseases or RNAs, such as employing transfer learning from large-scale biological networks or incorporating knowledge graph embeddings to infer features of novel nodes, thereby improving generalization. In addition, the ceRNA associations predicted by CERDA exhibit strong biological plausibility, suggesting their potential use as novel biomarkers or therapeutic targets in disease diagnosis and treatment.

In summary, CERDA offers a biologically meaningful and methodologically robust framework for modeling ceRNA–disease associations. Its application to cardiovascular disease, acute myeloid leukemia, and papillary thyroid cancer illustrates its effectiveness and generalizability across diverse biomedical contexts. By leveraging high-order topological features, this study offers new insights into the regulatory functions of mRNAs, lncRNAs, and miRNAs in complex diseases, and lays a foundation for future advancements in biomarker discovery and personalized medicine.

**Key Points**

- **Integrated ceRNA network construction**: captures multilevel regulatory interactions and high-order topological features from heterogeneous molecular datasets.
- **Multilayer graph convolutional network with high-order attention**: aggregates both direct and high-order neighbors to enhance regulatory relationship modeling.
- **High-order negative sampling strategy**: improves training efficiency and generalization by leveraging chi-subnode similarity during negative sample generation.

## Data Availability

The ceRNA network data are available at http://bio-bigdata.hrbmu.edu.cn/LncACTdb through the LncACTdb database, which provides comprehensive information on ceRNA interactions for biomedical research. Our source code is available at https://github.com/Chai-RAn6/CERDA-HOSR.
